# Changes in subclass-specific IgG Fc glycosylation associated with the postnatal maturation of the murine immune system

**DOI:** 10.1038/s41598-020-71899-7

**Published:** 2020-09-17

**Authors:** Gabriela Barrientos, Siniša Habazin, Mislav Novokmet, Yahia Almousa, Gordan Lauc, Melanie L. Conrad

**Affiliations:** 1 Laboratory of Experimental Medicine, Hospital Alemán, Buenos Aires, Argentina; 2 National Scientific and Technical Research Council (CONICET), Buenos Aires, Argentina; 3 Glycoscience Research Laboratory, Genos Ltd., Zagreb, Croatia; 4 Laboratory of Molecular Tumor Pathology, Institute of Pathology, Charité-University Medicine Berlin, Corporate Member of Freie Universität Berlin, Humboldt-Universität Zu Berlin, and Berlin Institute of Health, Berlin, Germany; 5 Department of Biochemistry and Molecular Biology, Faculty of Pharmacy and Biochemistry, University of Zagreb, Zagreb, Croatia; 6 Division of Psychosomatic Medicine, Department of Internal Medicine, Charité-Universitätsmedizin Berlin, Corporate Member of Freie Universität Berlin, Humboldt-Universität Zu Berlin, and Berlin Institute of Health, Berlin, Germany; 7 Institute of Microbiology, Infectious Diseases and Immunology, Charité-University Medicine Berlin, Corporate Member of Freie Universität Berlin, Humboldt-Universität Zu Berlin, and Berlin Institute of Health, Hindenburgdamm 30, 12203 Berlin, Germany

**Keywords:** Glycobiology, Ageing, Antibodies

## Abstract

Early postnatal life is characterized by a critical time period in which the developing neonatal immune system transitions from passive immunity, induced by protective maternal antibodies, to the competence of a fully functioning immune system. The inflammatory capability of both maternal and neonatal antibodies is governed by N-linked glycosylation of the Fc region, and though this has been examined extensively in adults, there is currently little information regarding antibody glycosylation patterns during early postnatal life. To characterize the murine IgG Fc glycosylation profile during early life, we used nano-LC-ESI-Qq-TOF mass spectrometry analysis to assess subclass specific Asn-297 glycosylation patterns in the serum of BALB/c mice from 5–60 days of age. From birth to adulthood, we observed a decline in proinflammatory Fc glycosylation in all IgG subclasses. This was shown by significantly reduced agalactosylated and monogalactosylated structures combined with increased sialylation after weaning at 45 and 60 days of age. This information indicates that the transition between neonatal life and adulthood in mice is accompanied by reduction of inflammatory IgG antibodies. Our study contributes to a growing body of literature indicating the importance of IgG Fc glycosylation and its association with inflammation during different life stages.

## Introduction

Antibodies act as key players in the adaptive immune response by binding to specific antigens and facilitating their phagocytosis by innate immune cells. The ability to elicit such immunity is due to their unique Y-shaped structure, with two antigen-binding fragments (Fab) that mediate antigen recognition and the fragment crystallizable (Fc) region that is responsible for effector functions. The most abundant antibody class with the longest half-life is Immunoglobulin G (IgG), which comprises 75% of antibodies in human plasma and exerts an array of effector functions including antigen recognition, antibody-dependent cell cytotoxicity and activation of the complement system^1^.

**Figure 1:**
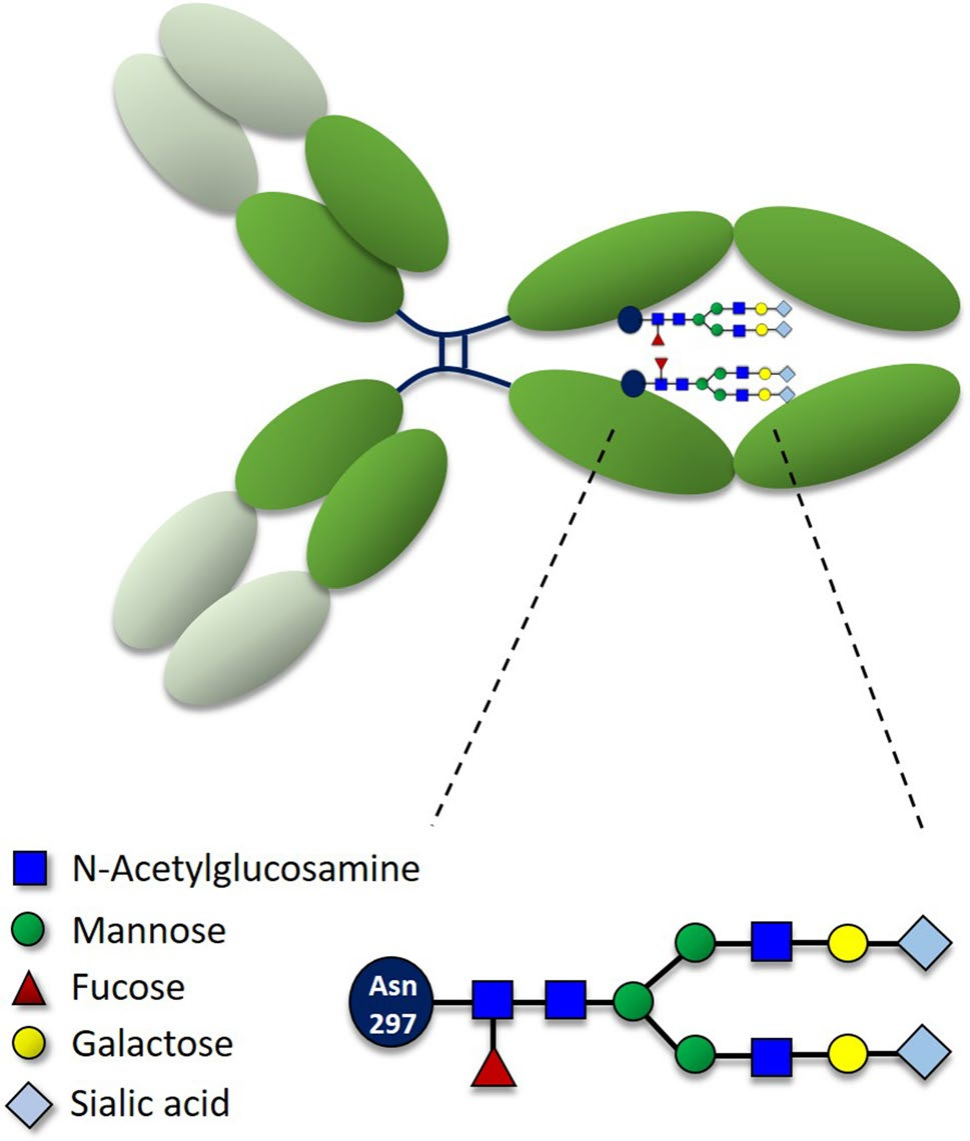
Diagram of the Asn-297 N-linked glycosylation in an IgG antibody. A diagram of an IgG molecule showing the two Fc glycosylation sites that influence antibody inflammatory capability. The glycan molecule consists of a core containing four *N*-acetylglucosamine residues (dark blue) and three branched mannose molecules (green). The core structure can be modified by the addition of a fucose (red), or galactose (yellow) and sialic acid (light blue). Sialylated mouse IgG glycans bear *N*-glycolylneuraminic acid (Neu5Gc) as sialic acid. The inset shows a biantennary, core-fucosylated, disialylated *N*-glycan (FA2G2S2).

Antibody function is largely dependent on the glycosylation status of the Fc region, which induces conformational changes that influence binding to Fc receptors (i.e., FcγRs) and C-type lectins^2,3^. IgG molecules display a single N-linked glycan attached to Asn-297 of their heavy chains, which directs antibody functionality^4^. The core *N*-glycan structure is built by two *N*-acetylglucosamine (GlcNAc) and three mannose residues (Fig. 1). This core structure can be modified through: the addition of fucose to the innermost GlcNAc, addition of the antennae by GlcNAc binding to mannose residues, extension of the antennae with galactose, sialylation of these additional galactose units or addition of a bisecting GlcNAc to the β-linked mannose on the trimannosyl core. In particular, the addition of terminal galactose or sialic acid residues to the core Fc glycan has raised much attention as it has been shown to promote anti-inflammatory effects^3,5–7^ (Fig. 1).

Changes to antibody glycosylation status are associated with many different pathophysiological states. IgG antibodies display decreased galactosylation and sialylation during periods of disease exacerbation in both humans and mice. This is seen in inflammation-mediated immune conditions such as rheumatoid arthritis^8,9^ inflammatory bowel disease^10,11^ and asthma^12,13^. Though these pro-inflammatory IgG alterations were noticed in rheumatoid arthritis patients over 30 years ago^14^, the importance of glycosylation as a determinant of antibody functionality has been recognized only recently.

Reminiscent of the glycosylation changes observed during disease pathogenesis, age-dependent accumulation of agalactosylated IgG molecules is also of particular interest. Referred to as IgG-G0, agalactosylated IgG has been suggested to contribute to the increased pro-inflammatory status associated with the aging process^15^. In this context, characterization of the IgG glycome in the steady state is highly relevant for identifying changes associated with pathological settings. While the pro-inflammatory changes associated with aging have been extensively characterized in humans from birth to senescence^14,16–20^, much less is known about glycosylation in mice, which are often used to model disease pathogenesis. Specifically, information is still lacking regarding IgG glycosylation status in early life. The first two months of postnatal development represent a critical window of immune system maturation^21^, and during this sensitive time period the presence of pro- or anti-inflammatory IgG molecules could substantially influence the establishment of full immune competence. Thus, our aim for the present study was to characterize the subclass-specific Fc glycovariations in the serum IgG pool associated with this period of postnatal development in BALB/c mice.

## Results

### Postnatal development is associated with an age-dependent increase of IgG1 galactosylation and sialylation

**Figure 2:**
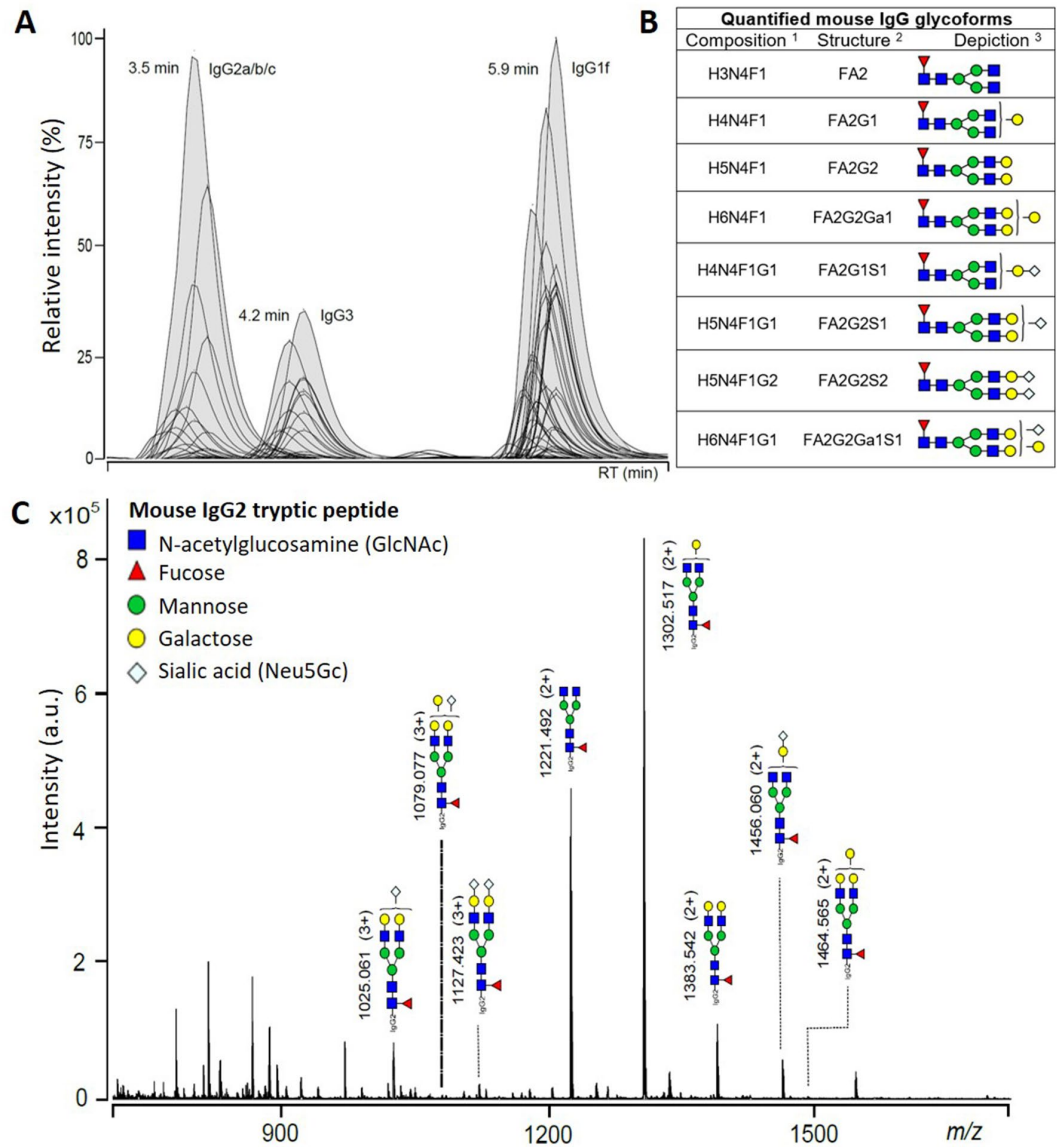
(**A**) Representative extracted ion chromatogram of BALB/c mouse IgG glycopeptides originating from five subclasses as separated on a C_18_ nano-column into three peaks. IgG2a/b/c glycopeptides cannot be separated in a short LC run and are treated as a whole. Allelic variant IgG1f is dominant for this strain and differs from IgG1i by amino acid substitution (phenylalanine to isoleucine). (**B**) Eight glycoforms reliably quantified for each IgG subclass with corresponding glycan compositions, structures and depictions. ^1^Composition: *H* hexose, *N* GlcNAc, *F* fucose, *G* Neu5Gc. ^2^Structure: *F* core fucose, *An* number of antennas, *Gn* number of galactoses, *Ga* galactoses linked by alpha linkage, *Sn* number of sialic acids (Neu5Gc). ^3^Depiction: blue square—GlcNAc, red triangle—core fucose, green circle—mannose, yellow circle—galactose, light blue diamond—sialic acid (Neu5Gc). (**C**) Summed mass spectrum of IgG2a/b/c peak with annotated doubly (M + 2H)^2+^ and triply (M + 3H)^3+^ charged glycopeptides.

**Figure 3:**
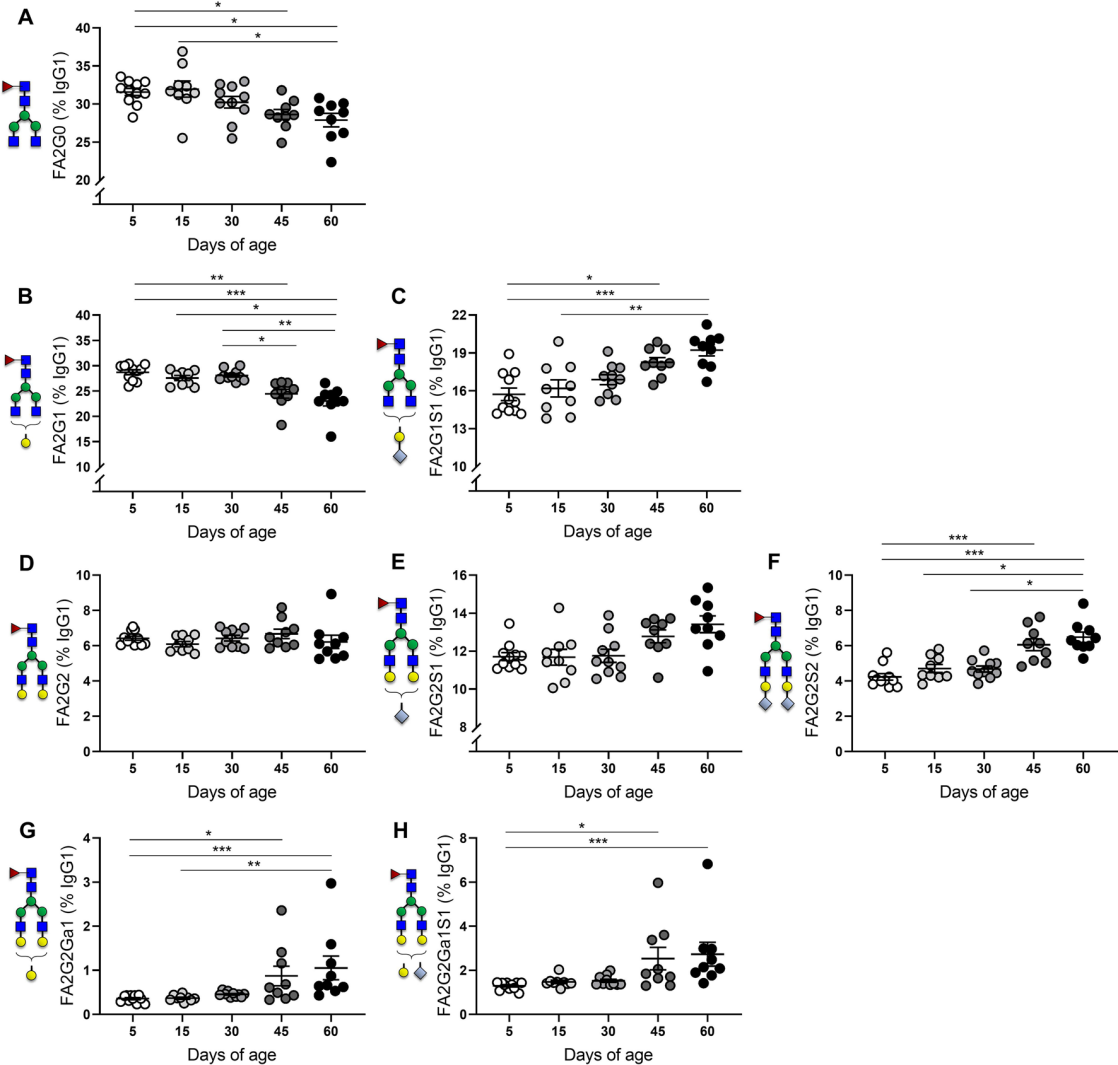
Mouse serum IgG1 Asn-297 glycosylation from 5–60 days of age. Percent galactosylation and sialylation of IgG1- (**A**) FA2G0, (**B**) FA2G1, (**C**) FA2G1S1, (**D**) FA2G2, (**E**) FA2G2S1 (**F**) FA2G2S2 (**G**) FA2G2Ga1 (**H**) FA2G2Ga1S1. Means ± SEM are shown. Male and female offspring age: 5 days (n = 11), 15 days (n = 9), 30 days (n = 10), 45 days (n = 9), 60 days (n = 9). Results represent two independently performed experiments. Significance is represented by *P < 0.05, **P < 0.01, ***P < 0.001, ANOVA or Kruskal–Wallis with Dunnett's multiple comparisons test.

To characterize the Asn-297 glycovariation in murine IgG1 during the progression from birth to adulthood, we analysed the frequency distribution of galactosylated and sialylated species during the first two months of postnatal development. A representative mass spectrum of the results and the characterized structures is displayed in Fig. 2. As shown in Fig. 3, we observed a progressive decline of agalactosylated (IgG1-FA2G0) and monogalactosylated (IgG1-FA2G1) IgG1 with age. Proinflammatory G0, representing ~ 30% of total IgG1 throughout the postnatal period analyzed, showed no variations during lactation stages (postnatal day (PN)5 and PN15) but progressively decreased post-weaning with significantly reduced frequencies at PN45 and PN60 (Fig. 3A). Though less pronounced, monogalactosylated structures (also representing ~ 30% of total IgG1) showed a similar pattern of variation, with no differences until PN30 but a significantly decreased percentage at PN45 compared to PN5 (Fig. 3B). Interestingly, sialylation of -G1 structures was markedly increased during progression to adulthood, showing significantly increased percentages at PN45 and PN60 (Fig. 3C). In contrast, we observed no age-dependent differences in the percentage of digalactosylated (IgG1-FA2G2) species, which accounted for less than 10% of total IgG1 (Fig. 3D). However, similarly to monogalactosylated IgG1, sialylation of digalactosylated structures was increased with progression to adulthood (Fig. 3E–F) with significantly increased percentages of IgG2-FA2G2S2 at PN45 and PN60 (Fig. 3F). The percentages of IgG1-FA2G2Ga1 glycoforms showed no differences during the first month of development but later increased significantly at PN45 and PN60 (Fig. 3G), with sialylated structures (FA2G2Ga1S1) displaying a similar pattern of variation with age (Fig. 3H). Of note, during the postnatal period analysed in our study, male and female offspring showed no differences in the kinetics of IgG1 glycosylation (see Supplementary Fig. 1).

### IgG2 glycosylation shifts towards an anti-inflammatory phenotype at 30 days of age

**Figure 4:**
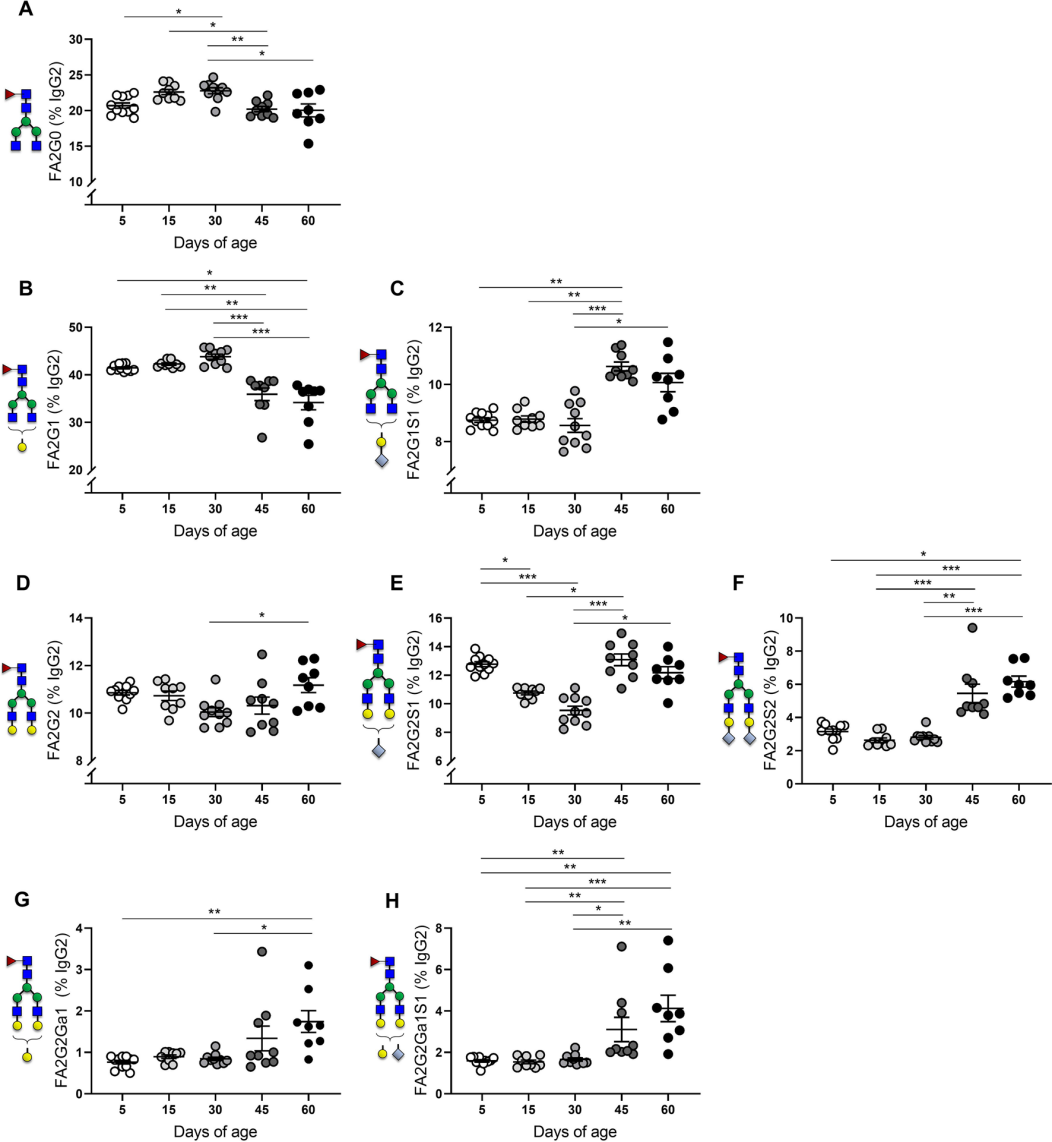
Mouse serum IgG2 Asn-297 glycosylation from 5–60 days of age. Percent galactosylation and sialylation of IgG2- (**A**) FA2G0, (**B**) FA2G1, (**C**) FA2G1S1, (**D**) FA2G2, (**E**) FA2G2S1 (**F**) FA2G2S2 (**G**) FA2G2Ga1 (**H**) FA2G2Ga1S1**.** Means ± SEM are shown. Male and female offspring age: 5 days (n = 11), 15 days (n = 9), 30 days (n = 10), 45 days (n = 9), 60 days (n = 9). Results represent two independently performed experiments. Significance is represented by *P < 0.05, **P < 0.01, ***P < 0.001, ANOVA or Kruskal–Wallis with Dunnett's multiple comparisons test.

The analysis of IgG2 Asn-297 glycovariants revealed a progressive increase of pro-inflammatory species during the first month of postnatal development, followed by a significant decline at PN45 and PN60 (Fig. 4). Both FA2G0 and FA2G1 frequencies peaked at PN30, but were significantly decreased later at PN45 and PN60 (Fig. 4A,B). This trend was associated with a marked increase in sialylation at PN45 (Fig. 4C), indicating a shift towards an anti-inflammatory phenotype. Interestingly, the kinetics of IgG2-FA2G2 frequencies showed an opposite trend, reaching minimum levels at PN30 and later increasing towards PN60 (Fig. 4D). This increase in G2 was linked with a significant increase in the frequencies of mono- and disialylated structures FA2G2S1 (Fig. 4E) and FA2G2S2 (Fig. 4F) starting at PN45. The anti-inflammatory shift in IgG2 glycovariants was also verified in the frequency distribution of α-1,3-galactosylated (α-gal) structures, which showed no changes until PN30 and were later significantly increased with increasing sialic acid content at PN45 and PN60 (Fig. 4G,H). Similarly to IgG1, the changes in IgG2 glycosylation did not differ between female and male offspring (Supplementary Fig. 2).

### Sialylation of IgG3 increases with progression to adulthood

**Figure 5:**
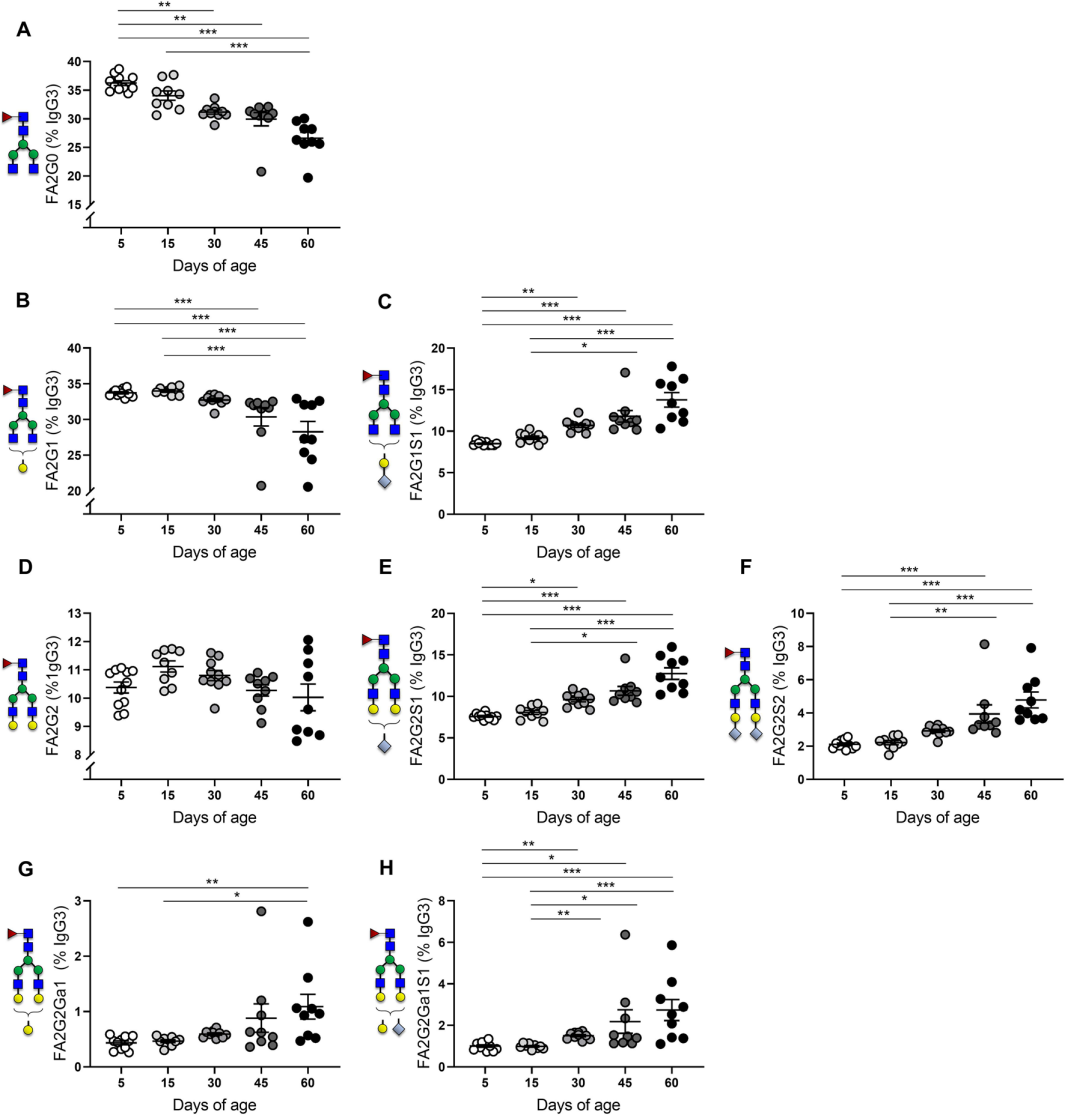
Mouse serum IgG3 Asn-297 glycosylation from 5–60 days of age. Percent galactosylation and sialylation of IgG3- (**A**) FA2G0, (**B**) FA2G1, (**C**) FA2G1S1, (**D**) FA2G2, (**E**) FA2G2S1 (**F**) FA2G2S2 (**G**) FA2G2Ga1 (**H**) FA2G2Ga1S1**.** Means ± SEM are shown. Male and female offspring age: 5 days (n = 11), 15 days (n = 9), 30 days (n = 10), 45 days (n = 9), 60 days (n = 9). Results represent two independently performed experiments. Significance is represented by *P < 0.05, **P < 0.01, ***P < 0.001, ANOVA or Kruskal–Wallis with Dunnett's multiple comparisons test.

Our analysis for IgG3 also revealed an anti-inflammatory trend with advancing age, characterized by an increased sialic acid content of the Asn-297 glycan (Fig. 5). Pro-inflammatory FA2G0 species declined post-weaning, showing significantly decreased frequencies from PN30 until PN60 (Fig. 5A). A similar kinetics were observed for FA2G1, with levels decreasing significantly starting at PN45 (Fig. 5B). Of note, this decline was accompanied by a progressive increase of FA2G1S1 structures, starting at PN30 (Fig. 5C). Digalactosylated species showed no age-dependent changes during postnatal development, but their sialic acid content increased significantly in the post-weaning period. Indeed, compared to PN5, significantly increased abundances of FA2G2S1 and FA2G2S2 were observed starting at PN30 (Fig. 5E) and PN45 (Fig. 5F) respectively. Likewise, the frequency of anti-inflammatory α-gal species showed no significant differences until PN60 (Fig. 5G), but displayed a similar kinetics of sialylation with significantly increased levels of -G2Ga1S1 from PN30 to PN60 (Fig. 5H). Male and female offspring displayed no differences in the frequency distribution of IgG3 glycovariants (Supplementary Fig. 3).

## Discussion

We analysed subclass specific IgG Fc glycosylation patterns in mice from infancy to adulthood (5–60 days of age). Our study demonstrated that during postnatal development, IgG1 and IgG3 subclasses were characterized by a steady decrease in proinflammatory agalactosylated and monogalactosylated structures with increasing age, with concurrent increases in alpha-1,3-galactosylated IgG in all antibody subclasses. IgG2 showed a slightly different pattern with mono- and agalactosylated structures increasing marginally from 5–30 days of age, and then significantly decreasing after weaning. Our results are in agreement with human studies showing that children between the ages of 6 and 25 years showed decreasing agalactosylated and increasing digalactosylated total IgG glycans corresponding with increasing age^19,22,23^. From adulthood onwards, human studies identify a reversal of this pattern at approximately age 25, proceeding to continuous rise in agalactosylated IgG which is thought to contribute to the aging process^15,16,18–20^.

The postnatal period represents a critical developmental window during which offspring rely on passive immunity to protect against infection, until the establishment of full immune competence^21,24^. In addition to increasing galactosylation, our analysis also revealed significant increases in sialylation in all murine IgG subclasses from infancy to adulthood, indicating that the postnatal maturation of the humoral immune response is associated with a shift towards a less pro-inflammatory profile of antibody glycosylation. Thus, these changes are likely associated with the offspring’s transition from passive immunity to endogenous IgG synthesis, which starts around the 4th week of age^25^. In our study, the IgG glycosylation measured at PN5 and PN15 likely reflects direct IgG transfer from the maternal breast milk, which occurs until approximately PN16 and is mediated by expression of the neonatal Fc receptor (FcRn) in the murine gut^26^. Since IgG has a 21 day half-life^27^, the later time points in our study (PN30 to PN60) represent transition stages from the onset of intrinsic IgG production mingled with decreasing remnants of maternally transferred antibodies. This is strongly supported by our data, as during exclusive breastfeeding at the age of 5 and 15 days, we observed no differences in IgG glycosylation patterns with the exception of IgG2-FAG2S1.

The differential kinetics of glycosylation observed for murine IgG subclasses in our study may be related to subclass-specific differences in maternal transfer (i.e., differential rates of transplacental transfer versus intestinal passage via breastfeeding). Considering placental IgG transfer, though it has been shown in humans that IgG Fc sialylation increases during pregnancy^28^ it is also known that IgG glycosylation plays a role in the preferential placental transfer of antibodies^29^. In mice, pioneering studies on the mechanisms driving passive immunity have shown that while murine IgG1 is exclusively transferred via breast milk^30^, IgG3 is mostly acquired prenatally across the murine yolk sac whereas transfer of IgG2 involves both routes^30,31^. Before further conclusions can be drawn regarding how glycosylation influences the maternal contribution of IgG antibodies through the placenta and breast milk, more murine studies are needed. Finally, another factor that should be taken into account is that glycosylation per se can influence the clearance rate of a particular IgG subclass. For example, it has been shown that mouse agalactosylated IgG2a exhibits a 20–40% longer half-life in circulation than its fully galactosylated counterparts^32^. Regarding the kinetics of IgG2 glycosylation observed in our study, the more pronounced shift towards an anti-inflammatory profile starting at PN30 may reflect the different functional properties ascribed to each IgG subclass.

Other murine studies examining subclass specific differences in IgG glycosylation have uniformly reported that the most contrasting changes are observed between the major subclasses IgG1 and IgG2, with a predominance of agalactosylated structures in IgG1 and increased terminal galactose and sialic acid content for IgG2 glycoforms^33,34^. In mice, these two subclasses contrast in their effector functions, with IgG1 being predominantly an immunoregulatory, Th2-associated immunoglobulin and IgG2 displaying a potent pro-inflammatory effect due to its preferential association with activating Fc receptors^35,36^. In this context, the rapid shift of IgG2 glycosylation towards increased terminal galactosylation and sialylation observed in our study, concomitant with the onset of intrinsic antibody production might constitute a protective mechanism to dampen potentially harmful immune hyperresponsiveness associated with IgG2 effector functions.

Our data provides important information regarding IgG Fc glycosylation changes from infancy to adulthood in Balb/c mice. Caution should be taken, however, when extrapolating this data to other mouse strains, as de Haan et al. found marked differences in IgG glycosylation between BALB/c and C57BL/6 mice^37^. This raises the compelling idea that IgG glycosylation may play a role in the differences in inflammation commonly observed in these two mouse strains. Indeed, it is well known that immune responses in BALB/c mice are more dominated by a Th2 cytokine profile, whereas C57BL/6 mice are more skewed toward pro-inflammatory Th1 responses^38^. To date, contrasting results have been reported regarding sex-dependent differences in murine IgG glycosylation. While our study is in agreement with the lack of differences previously reported in adult mice by de Haan^37^, other reports have demonstrated lower levels of bisection and sialylation and increased galactosylation in females than in males^39^. Human studies have also found substantial variation in IgG Fc glycosylation in women, particularly during puberty and nearing menopause^22,23^. This highlights a possible hormonal role in IgG glycosylation that should be studied in more detail, particularly in light of previous studies showing an association between IgG galactosylation and sialylation and steroid hormone signaling^40,41^. The divergent results regarding sex differences in IgG glycosylation reported in mouse studies might be attributed to technical variation (i.e., use of different analytical methods detecting different sets of glycoforms) between studies or more importantly, as in the present study, related to age since it is expected that steroid hormone regulation would not exert a significant influence on IgG glycosylation before puberty (which in most mouse strains is reached at 6–8 weeks of age).

Our study observed age-dependent differences in Fc glycosylation between murine antibody subclasses from birth to adulthood, which can be correlated with the postnatal developmental window characterizing the transition from passive immunity to full immune competence. Since antibody function is not only determined by its affinity to specific Fc receptors but also by Fc glycosylation, analysis of glycosylation in different antibody subclasses in the steady-state is an absolute pre-requisite for the identification of changes associated to disease pathogenesis. In particular, given that mouse models are being increasingly used in the study of disease progression and for safety assessments of new therapeutic agents, the data reported herein becomes of critical importance for the design of translational studies related to developmental immunotoxicity and the design of immune interventions and vaccine strategies during early life. In recent years, the direct link between immune dysfunction and chronic disease has become increasingly evident. Therefore, a more comprehensive knowledge of critical windows of intervention during immune system development might help to reduce the incidence of later life chronic disease.

## Methods

### Chemicals

Protein G sepharose fast flow was purchased from GE Lifesciences. Tris, glycine, ammonium bicarbonate and trifluoroacetic acid were purchased from Sigma-Aldrich. LC–MS grade acetonitrile was from J. T. Baker. Sequencing grade trypsin was from Promega Corporation, C_18_ beads used for glycopeptide cleanup from Macherey–Nagel, and ultrapure water was produced in-house by a Direct-Q 3 UV water purification system (Merck Millipore) and was of 18 MΩ or better.

### Animals

12-week-old female BALB/c mice were obtained from Janvier Labs (Le Genest-Saint-Isle, France), and housed four animals per cage in a 12/12 h light/dark cycle. Food and water were available ad libitum. All animal experiments were approved by the ethics committee of the local authority Landesamt für Gesundheit und Soziales (LAGeSo) (authorized to approve the experiments on animals), and were performed in accordance with German and international guidelines.

### Experimental design

BALB/c male and female mice were mated and visualization of a mucous plug was considered gestation day (G)0. Plugged female mice were removed from the mating cage and housed together until G17, upon which they were housed singly to give birth. At postnatal day (PN)5 and PN15, blood was collected from pups that were euthanized by decapitation or cervical dislocation respectively. The remaining pups were weaned at PN21, then at 30, 45 and 60 days of age blood was collected from euthanized offspring for antibody glycosylation analysis.

### Serum collection and IgG purification

Blood was stored at room temperature for 1 h, centrifuged and the serum was stored at − 80 °C. For purification, Protein G affinity beads were used to capture IgG from 15 µl of serum. Proteins interacted with the beads while shaken for 1.5 h, after which the beads were washed seven times with 1.5 ml PBS. IgG was eluted with 100 μl of 100 mM formic acid and eluates were dried for 2 h in a vacuum concentrator at 60 °C^13^.

### Antibody glycosylation analysis

Subclass-specific mouse IgG N-linked glycosylation analysis was performed as previously described^33^. Briefly, dried IgG was dissolved in 40 µl 25 mM ammonium bicarbonate and 0.2 µg of sequencing grade trypsin was added. After incubation for 18 h at 37 °C, obtained glycopeptides were desalted on C_18_ beads, dried in a vacuum centrifuge and redissolved in 40 µl of ultrapure water. Nano-LC-ESI-Qq-TOF analysis was performed on a Compact time-of-flight mass spectrometer (Bruker, Billerica, USA) operated in reflectron positive mode by injecting 5 µl of sample, and separation was achieved on a HALO C_18_ nano-LC column (150 × 0.1 mm, 5 µm, 90 Å, Advanced materials technology, Wilmington, USA). The high-throughput LC–MS method used for Fc-specific mouse IgG glycosylation analysis has already been described elsewhere^33^. Spectra were acquired in *m/z* range 50–3500 at a rate of 2 × 0.5 Hz. For data analysis, files were first converted to .mzXML format using ProteoWizard msConvert^42^ (v. 3.0) and processed with LaCy Tools software^43^ (v. 1.0.1). Manual data curation was then performed to exclude analytes with low mass accuracy, signal-to-noise ratio and isotopic pattern quality from further quantitation. Glycopeptide peak areas of doubly and triply charged ions observed as proton adducts were summed and normalized by total area for each subclass detected. A detailed description of the LaCy Tools parameters used for data extraction together with detected glycoforms, theoretical *m*/*z* values and manual data curation criteria is given in Supplementary Table 1.

### Statistical analysis

All samples were tested for normal distribution using a Shapiro-Wilk normality test. Parametric data were analysed using one-way ANOVA and non-parametric data with a Kruskal–Wallis test, followed by Dunnett’s multiple comparisons test (all groups compared with each other). Data are presented as mean ± SEM. Calculations were performed with GraphPad Prism 8 software^13^.

## Supplementary information

Supplementary Figures.

Supplementary Legend.

Supplementary Tables.

## Data Availability

Raw MS1 data files (.d) are publicly available and deposited in PeptideAtlas database under data set identifier: PASS01606 (https://www.peptideatlas.org/PASS/PASS01606).
