# Supplementary material for: Changes in subclass-specific IgG Fc glycosylation associated with the postnatal maturation of the murine immune system
Source: Sci Rep. 2020 Sep 17;10:15243. doi: 10.1038/s41598-020-71899-7 (PMC7498460; doi:10.1038/s41598-020-71899-7)
Supplement: Supplementary file 1 — Supplementary Figures. [file 41598_2020_71899_MOESM1_ESM.docx]

**Title: Changes in subclass-specific IgG Fc glycosylation associated with the postnatal maturation of the murine immune system**

Gabriela Barrientos^1,2^, Siniša Habazin^3^, Mislav Novokmet^3^, Yahia Almousa^4^, Gordan Lauc^3,5^, Melanie L. Conrad^6,7^*

**Supplementary Figures**

**
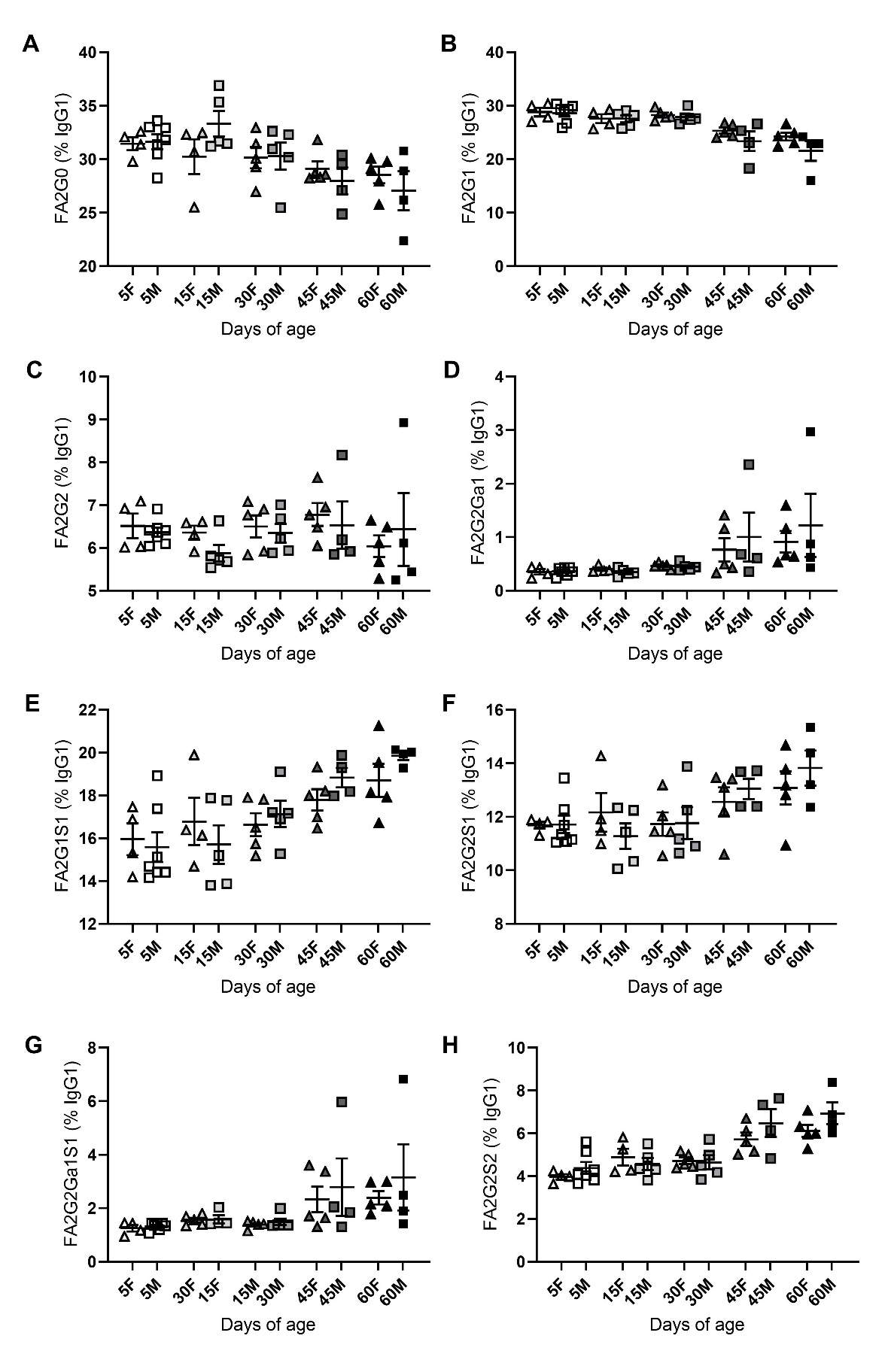
**

**Supplementary Figure 1: Serum IgG1 Fc glycosylation does not significantly differ between male and female mice.**

Percent IgG galactosylation and sialylation of in mice from 5-60 days of age. Females (F), Males (M). IgG1- (**A)** FA2G0, **(B)** FA2G1, **(C)** FA2G2, **(D)** FA2G2Ga1, **(E)** FA2G1S1 **(F)** FA2G2S1 **(G)** FA2G2Ga1S1 **(H)** FA2G2S2**.** Means ± SEM are shown. Male and female offspring age: 5 days (n = 11), 15 days (n = 9), 30 days (n = 10), 45 days (n = 9), 60 days (n = 9). Results represent two independently performed experiments. Significance is represented by **P* < .05, ***P* < .01, ****P* < .001, ANOVA or Kruskal-Wallis with Dunnett's multiple comparisons test.

**
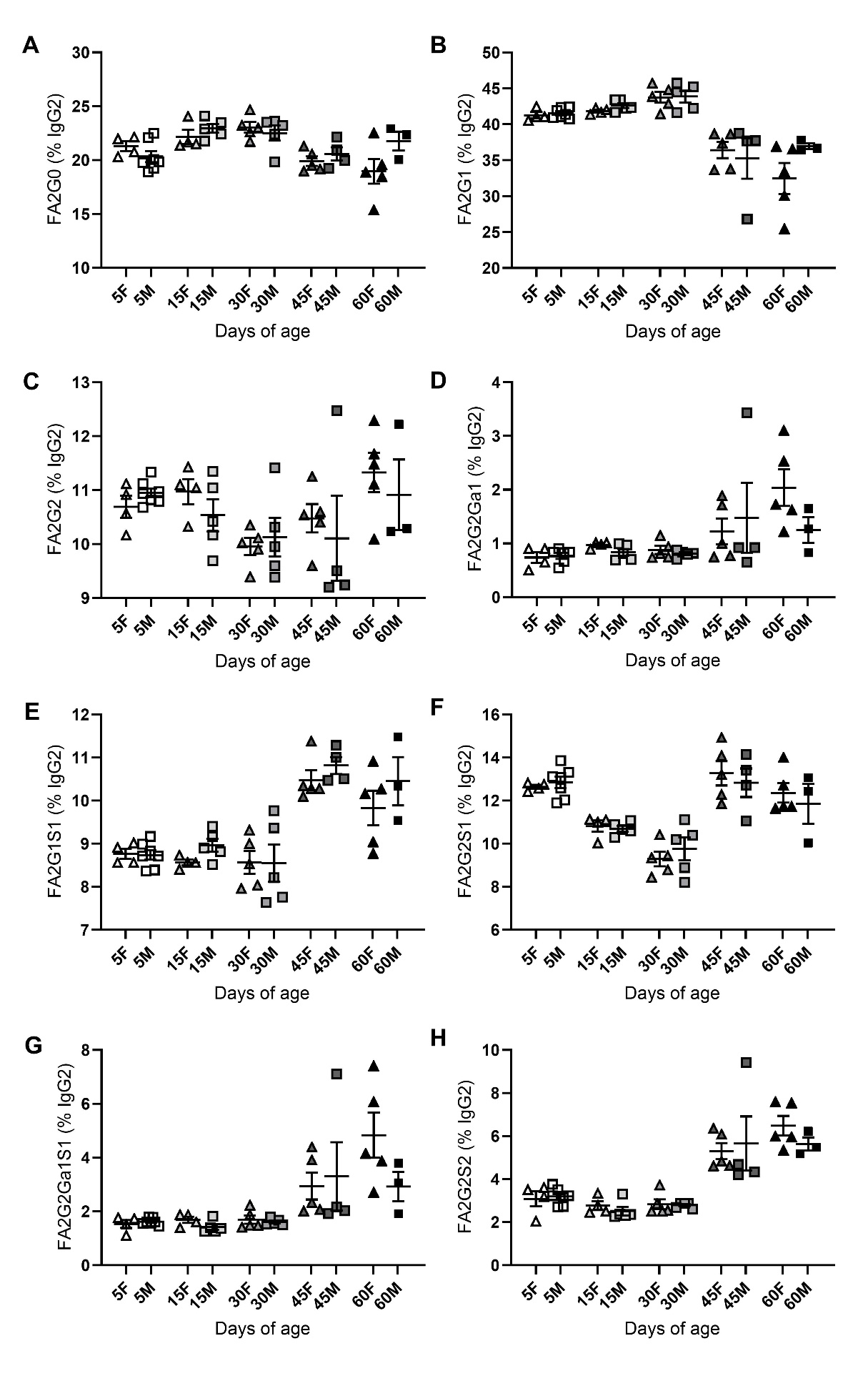
**

**Supplementary Figure 2: Serum IgG2 Fc glycosylation does not significantly differ between male and female mice.**

Percent IgG galactosylation and sialylation of in mice from 5-60 days of age. Females (F), Males (M). IgG2- (**A)** FA2G0, **(B)** FA2G1, **(C)** FA2G2, **(D)** FA2G2Ga1, **(E)** FA2G1S1 **(F)** FA2G2S1 **(G)** FA2G2Ga1S1 **(H)** FA2G2S2**.** Means ± SEM are shown. Male and female offspring age: 5 days (n = 11), 15 days (n = 9), 30 days (n = 10), 45 days (n = 9), 60 days (n = 9). Results represent two independently performed experiments. Significance is represented by **P* < .05, ***P* < .01, ****P* < .001, ANOVA or Kruskal-Wallis with Dunnett's multiple comparisons test.

**
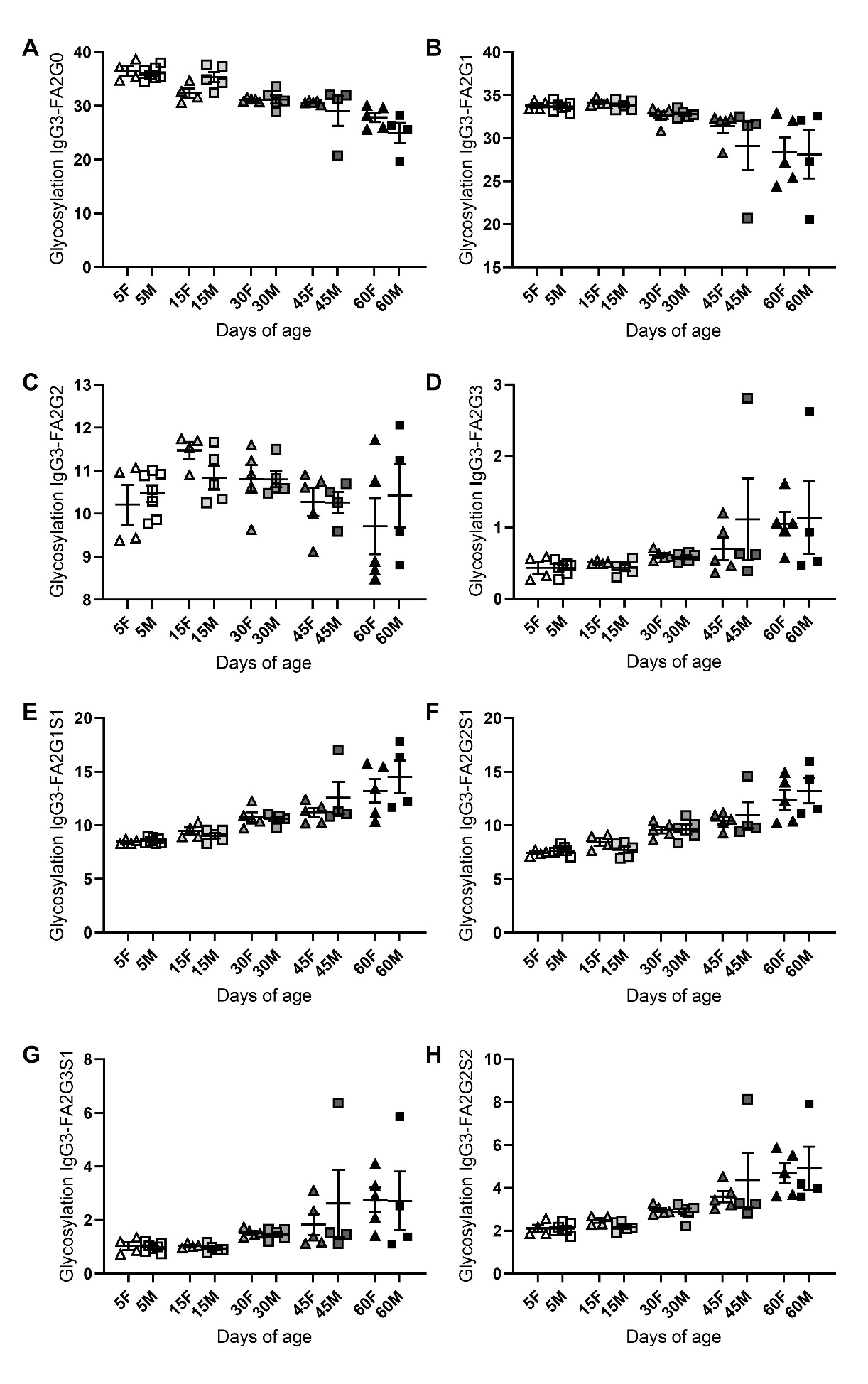
**

**Supplementary Figure 3: Serum IgG1 Fc glycosylation does not significantly differ between male and female mice.**

Percent IgG galactosylation and sialylation of in mice from 5-60 days of age. Females (F), Males (M). IgG3- ((**A)** FA2G0, **(B)** FA2G1, **(C)** FA2G2, **(D)** FA2G2Ga1, **(E)** FA2G1S1 **(F)** FA2G2S1 **(G)** FA2G2Ga1S1 **(H)** FA2G2S2**..** Means ± SEM are shown. Male and female offspring age: 5 days (n = 11), 15 days (n = 9), 30 days (n = 10), 45 days (n = 9), 60 days (n = 9). Results represent two independently performed experiments. Significance is represented by **P* < .05, ***P* < .01, ****P* < .001, ANOVA or Kruskal-Wallis with Dunnett's multiple comparisons test.
