# Supplementary material for: Changes in subclass-specific IgG Fc glycosylation associated with the postnatal maturation of the murine immune system
Source: Sci Rep. 2020 Sep 17;10:15243. doi: 10.1038/s41598-020-71899-7 (PMC7498460; doi:10.1038/s41598-020-71899-7)
Supplement: Supplementary file 2 — Supplementary Legend. [file 41598_2020_71899_MOESM2_ESM.docx]

**Supplementary Table 1**

**(A)**Theoretical *m*/*z* values of doubly and triply charged detected glycopeptides from mouse IgG for subclasses IgG1, IgG2a/b/c and IgG3. (**B)** Detailed description of different alignment, calibration and extraction parameters for LaCy Tools high throughput glycoproteomic data processing package with additional references on the software. (**C)** Original data summary as exported by LaCy Tools including sample names and parameter settings used. (**D)** Manual data curation for [M+2H]^2+^ including a list of data curation criteria. **(E)** Manual data curation for [M+3H]^3+^. **(F)** Summed absolute areas for eight quantified mouse IgG glycoforms per each subclass. **(G)** Relative areas for eight quantified mouse IgG glycoforms per each subclass.
